# Intake of Pyriproxyfen Through Contaminated Food by the Predator *Ceraeochrysa claveri* Navás, 1911 (Neuroptera: Chrysopidae): Evaluation of Long-Term Effects on Testes via Transcriptome Analysis †

**DOI:** 10.3390/insects16060567

**Published:** 2025-05-28

**Authors:** Jefferson Fogaça Tomacheski, Ana Silvia Gimenes Garcia, Rafael Takahiro Nakajima, Fábio Malta de Sá Patroni, Elton Luiz Scudeler, Rafael Henrique Nóbrega, Daniela Carvalho dos Santos

**Affiliations:** 1 Laboratory of Insects, Department of Structural and Functional Biology, Institute of Biosciences, São Paulo State University—UNESP, Botucatu 18618-689, SP, Brazil; j.tomacheski@unesp.br (J.F.T.); ana.garcia@unesp.br (A.S.G.G.); 2 Laboratory of Molecular and Reproductive Biology, Department of Structural and Functional Biology, Institute of Biosciences, São Paulo State University—UNESP, Botucatu 01049-010, SP, Brazil; takahiro.nakajima@unesp.br (R.T.N.); rafael.nobrega@unesp.br (R.H.N.); 3 Molecular Biology and Genetic Engineering, Institute of Biology, State University of Campinas—UNICAMP, Campinas 13083-875, SP, Brazil; fabio.m.patroni@gmail.com; 4 Department of General and Applied Biology, Institute of Biosciences, São Paulo State University—UNESP, Rio Claro 13506-900, SP, Brazil; elton.scudeler@unesp.br; 5 Electron Microscopy Center, Department of Structural and Functional Biology, Institute of Biosciences, São Paulo State University—UNESP, Botucatu 18618-689, SP, Brazil

**Keywords:** green lacewing, insecticide, sublethal effects, RNA-seq, testis

## Abstract

Understanding the sublethal effects of insecticides on non-target insects is essential for integrated pest management (IPM). This study aimed to evaluate the differentially expressed genes (DEGs) in the testes of *Ceraeochrysa claveri* adults exposed to pyriproxyfen during the larval stage. Larvae (0–12 h) were fed *Diatraea saccharalis* eggs treated with pyriproxyfen (50 and 100 mg a.i. L^−1^) for 10 days. After this exposure, the larvae were fed untreated eggs until pupation. The testes from the adults were extracted for RNA extraction, library construction, and sequencing. The reads were de novo assembled, and the genes annotated based on their ORF homology. A total of 46 DEGs were identified for the 50 mg a.i. L^−1^ vs. control, 47 DEGs for the 100 mg a.i. L^−1^ vs. control, and 64 DEGs for 50 mg vs. 100 mg a.i. L^−1^ treatments. To validate the DEGs through RT-qPCR, the genes BPHL, Large2, MLX, and Talin-1 were selected. The results indicate that the exposure of *C. claveri* larvae to pyriproxyfen could alter the gene expression and lead to delayed effects in adults. This study provided a novel approach for assessing the sublethal effects of pyriproxyfen *C. claveri* and contributed valuable information to enhance IPM strategies.

## Simple Summary

The green lacewing, *Ceraeochrysa claveri*, is an insect commonly utilized in integrated pest management (IPM) programs due to its predatory behavior during the larval stage, which helps control populations of pest insects. Understanding the sublethal effects of insecticides on non-target insects is crucial for effective IPM. This study aimed to evaluate the impact of pyriproxyfen on the gene expression in the testes of adult *C. claveri* exposed to insecticide during the larval stage. The larvae were fed *Diatraea saccharalis* eggs treated with pyriproxyfen for 10 days. After this, the larvae were fed untreated eggs until pupation. The testes from the adults were then extracted for molecular analysis. A transcriptome assembly was performed to identify differentially expressed genes (DEGs) between the three treatments analyzed. A total of 46 DEGs were identified in the first comparison, 47 DEGs in the second comparison, and 64 DEGs in the third comparison. Four genes (BPHL, Large2, MLX, and Talin-1) were selected for validation. The results indicated that the exposure of *C. claveri* larvae to pyriproxyfen could alter the gene expression and lead to delayed effects in adults. This study provided a novel approach for assessing the sublethal effects of pyriproxyfen on *C. claveri*, contributing valuable information to enhance IPM strategies.

## 1. Introduction

Neuropterans (Neuroptera Linnaeus, 1758) are cosmopolitan insects, with 6697 species described across sixteen extant families. Chrysopidae is the second-largest family within Neuroptera, comprising approximately 1486 species [1,2]. These insects play a crucial role in biological control as part of integrated pest management (IPM) programs due to their polyphagous feeding habits; they prey on aphids, phytophagous mites, leafhoppers, whiteflies, psyllids, and thrips, as well as the eggs and larvae of Lepidoptera, Coleoptera, and Diptera [3,4,5,6].

However, non-target insects coexist in the same environment as pests, and both groups are affected by the mode of action of insecticides. While various studies have documented the lethal effects of insecticides on non-target insects, fewer have investigated the sublethal effects on beneficial insects that survive exposure [7,8,9]. Among the available synthetic insecticides, pyriproxyfen is a juvenile hormone analog [10]. It interferes with the natural hormone system in insects, affecting embryonic development, metamorphosis, and causing sterility [11,12]. Molecular studies are used to analyze the effects of pyriproxyfen on target insects, helping evaluate its effectiveness in reducing target insects [13,14,15]. Conversely, molecular approaches are also employed to assess the extent of pyriproxyfen’s impact on non-target insects [16,17,18,19,20,21]. In chrysopids, also known as lacewings, pyriproxyfen has been shown to cause cellular damage in the testes, midgut, and fat body [22,23]. Despite these facts, these insects exhibit potential tolerances or resistances to several insecticides [24,25,26,27,28,29], allowing them to reproduce and sustain their populations in the field, which is advantageous for IPM strategies. Regardless of this, little is known about their genetic responses to such exposures.

As a molecular tool, transcriptome analysis (RNA-seq) enables the construction of a comprehensive transcript database, which is essential for understanding the diversity of mRNA [30]. Several studies that have focused on non-model insects have been conducted to obtain unbiased transcript profiles, allowing for a holistic analysis of insect physiology [31]. However, there have been few studies that have utilized transcriptomic analysis to address unresolved issues in chrysopid taxonomy [32] or to apply findings to agricultural contexts. For instance, *Chrysoperla sinica* (Tjeder) has been used to elucidate the molecular mechanisms of flight [33] and to identify candidates for olfactory genes through antennal transcriptome analysis [34]. *Chrysopa pallens* (Rambur, 1838) was exposed to the neonicotinoid nitenpyram (NIT) and acute toxicity was assessed by analyzing differentially expressed genes (DEGs) [35]. Additionally, chemoreception genes specific to this species were also identified [36]. Several cytochrome P450 genes in *Chrysoperla zastrowi sillemi* Esben-Petersen were found to be differentially expressed in response to imidacloprid [29]. Furthermore, *Chrysoperla carnea* Stephens was studied in relation to a biostable kinin peptide, demonstrating that IPM strategies could effectively integrate chrysopids and bioinsecticides [37].

*Ceraeochrysa* Adams is an important genus of green lacewings used in integrated pest management (IPM) programs in Brazil due to their high reproductive potential, resistance to various insecticides, and the voracious predatory behavior of their larvae [6]. Among these, *Ceraeochrysa claveri* (Navás, 1911) is a non-model insect employed in ecotoxicological studies to demonstrate the sublethal effects of insecticides on beneficial insects. Previous studies have shown that exposure to pyriproxyfen results in damage to the Malpighian tubules, midgut, fat body, cocoon spinning, and testes of *C. claveri* [22,23,38]. However, until now, there have been no transcriptomic data available to evaluate the molecular effects of insecticide exposure on this species. Since maintaining this beneficial insect in the field is desirable, developing improved IPM strategies to prevent population decline is essential. Given that previous studies confirmed that pyriproxyfen can cause morphological changes in testicular tissue [22], the aim was to evaluate its sublethal effects on gene expression in adult *C. claveri* using RNA-seq.

## 2. Materials and Methods

### 2.1. Insect Rearing

*C. claveri* larvae were reared in the Insect Laboratory at the Biosciences Institute, São Paulo State University (UNESP), Botucatu, SP, Brazil. The insect colony was maintained in a growth chamber under controlled environmental conditions: temperature of 25 ± 1 °C, relative humidity of 70 ± 10%, and a photoperiod of 12:12 h (light/dark). The larvae were fed ad libitum on *Diatraea saccharalis* (Lepidoptera: Crambidae) eggs until pupation. The adult insects were raised on an artificial diet composed of honey and brewer’s yeast (1:1). *D. saccharalis* eggs were supplied by CETMA Comércio de Agentes para Controle Biológico, Lençóis Paulista, SP, Brazil.

### 2.2. Bioassays

Newly hatched *C. claveri* larvae (0–12 h) were fed ad libitum on *D. saccharalis* eggs treated with two concentrations of the insecticide pyriproxyfen, which was used in its commercial formulation, Tiger 100 EC^®^ (Sumitomo Chemical do Brasil Representações Ltd., São Paulo, SP, Brazil). The insecticide was diluted in deionized water to concentrations of 50 mg a.i. L^−1^ and 100 mg a.i. L^−1^, representing 50% and 100% of the maximum field recommended concentrations (MFRCs) of pyriproxyfen for Brazilian crops, like soybean, coffee, tomato, and citrus, where chrysopids are commonly found [39].

*C. claveri* larvae were randomly divided into three experimental groups, with each group consisting of three replicates (*n* = 50 per replicate, totaling 150 insects per group). The larvae were individually placed in polyethylene pots. *D. saccharalis* eggs were treated with two dosages of pyriproxyfen using a dipping method: the egg clusters were immersed in the insecticide solution for 5 s, then dried at room temperature for 1 h. The treated eggs were provided ad libitum and replaced every three days, to prevent dehydration and maintain insecticide efficacy. The control group was fed eggs that had been immersed only in distilled water for 5 s and then dried at room temperature for 1 h [22,23,38]. All experimental groups were maintained under the same environmental conditions as the insect rearing.

Newly emerged adults from each experimental group were placed in polyethylene pots and fed on the previously described artificial diet. Afterward, only adult males aged 10 days (when they reach sexual maturity) were selected for testes extraction.

### 2.3. RNA Extraction, Library Preparation, and Sequencing

*C. claveri* adults (10 days old) were dissected, and the testes were collected in pools of 50 testes each. Three replicates were performed for each group (comprising a control group and two treated groups), which resulted in a total of 150 testes per group. The insects were cryoanesthetized and dissected from the dorsal region, then immersed in an insect saline solution (0.1 M NaCl; 0.1 M Na_2_HPO_4_; 0.1 M KH_2_PO_4_). The testes were immediately frozen in liquid nitrogen and stored in a biofreezer at −80 °C. RNA extraction and purification, library construction, and sequencing were conducted by NGS Soluções Genômicas (Piracicaba, SP, Brazil) using the Illumina platform (HiSeq 2000, 2 × 100). The raw sequence data have been deposited in the SRA under the accession code PRJNA1153524.

### 2.4. Reads Filtering, De Novo Assembly, and Annotation

A read quality analysis and the identification of highly representative sequences were conducted using FastQC v.0.12.0 [40]. The read quality was assessed (≥80%, phred value = 33), and adapter removal was performed using Trimmomatic v.0.39 [41]. Contaminants and rRNA were eliminated through a customized database of microorganisms and rRNA using the software BBduk v.38.90 [42].

The reads were assembled de novo with Trinity v.2.15.0 [43,44]. Redundant transcripts with ≥98% identity were removed using CD-HIT EST v.4.8.1 [45,46]. The final assembled transcripts underwent quality analysis and were subsequently used as a reference for read alignment with HISAT2 v.2.2.1 [47]. Additionally, a search for orthologous genes was performed using BUSCO v.5.4.7 [48,49] with the *arthropoda odb10* database (accessed on 20 March 2023).

Gene annotation was carried out by scanning the open reading frames (ORFs) for homology against the UniProt insect database and the Pfam database using TransDecoder v.5.7.0 [50] and Sma3s [51], both of which were run with default parameters.

### 2.5. Differentially Expressed Genes (DEGs) and Gene Ontology

The transcript count matrix was generated using the scripts align_and_estimate_abundance.pl and abundance_estimates_to_matrix.pl from Salmon [52]. Library analyses, TMM normalization, and the assessment of differentially expressed genes (DEGs) between the groups were conducted using the EdgeR package [53,54,55] in the R/Bioconductor environment v.4.1.2 [56]. Transcripts with a log2 fold change ≥2, log2 fold change ≤−2, and an FDR ≤ 0.05 were considered differentially expressed [57].

Additionally, a gene ontology analysis was performed for the DEGs against the Gene Ontology database [58]. The DEGs data were validated by RT-qPCR, as described below.

### 2.6. RT-qPCR for DEGs Validation

Four genes were randomly selected for the validation of differentially expressed genes (DEGs) using RT-qPCR. The analyses were conducted at the Molecular and Reproductive Biology Lab, Biosciences Institute, UNESP, Botucatu, SP, Brazil. Primers (listed in Table 1) were selected from the DEGs identified through RNA-seq analysis and were designed using Primer3Plus [59], PCR Primer Stats [60], and OligoCalc [61]. The total RNA used for the sequencing was used to synthesize cDNA with the iScript™ cDNA Synthesis Kit (Bio-Rad, Hercules, CA, USA).

RT-qPCR was performed using iTaq Universal SYBR Green Supermix (Bio-Rad, Hercules, CA, USA) on a StepOnePlus™ (Applied Biosystems™, Waltham, MA, USA) according to the manufacturer’s instructions. The reaction mix consisted of 10 µL total volume: 5 µL of SYBR qPCR premixture, 1 µL of each 10 µM primer, 2.5 µL of cDNA, and 0.5 µL of Milli-Q water. Three cDNA replicates, each with two technical replicates, were used for the samples (control, 50 mg a.i. L^−1^, and 100 mg a.i. L^−1^ treatments). The qPCR conditions were set as follows: 95 °C for 10 min, followed by 35 cycles of 95 °C for 15 s and 60 °C for 1 min.

To normalize the relative expression, the glyceraldehyde-3-phosphate dehydrogenase (GAPDH) gene was used as the housekeeping gene [62] while employing the 2^−ΔΔCT^ method [63]. The experiment was conducted in accordance with the MIQE guidelines [64]. One-way ANOVA was performed for comparisons between the groups using GraphPad Prism v.8.0.1.244.

## 3. Results

### 3.1. De Novo Assembly and Annotation

After sequencing, a total of 476,870,972 raw reads were generated. These reads were assessed for quality using FastQC and subsequently trimmed with Trimommatic and BBduk, which resulted in 448,210,677 filtered reads, which accounted for 94% of the original raw reads. The de novo assembly produced 260,417 transcripts, of which 172,686 were classified as genes. The assembly exhibited a GC content of 30.68%, an N50 length of 914 bp, and a median contig length of 370 bp.

The quality of the assembly demonstrated a 98.38% alignment of the reads against the transcriptome assembly used as a reference. In the BUSCO analysis, 1013 groups were searched for orthologous genes, where 991 groups matched, which represented 97.8% of the data. Among these, 630 groups were classified as complete and a single copy (62.2%), 361 groups as complete and duplicated (35.6%), 15 groups as fragmented (1.5%), and 7 groups were classified as missing (0.7%).

### 3.2. DEGs Analyses

**Figure 1:**
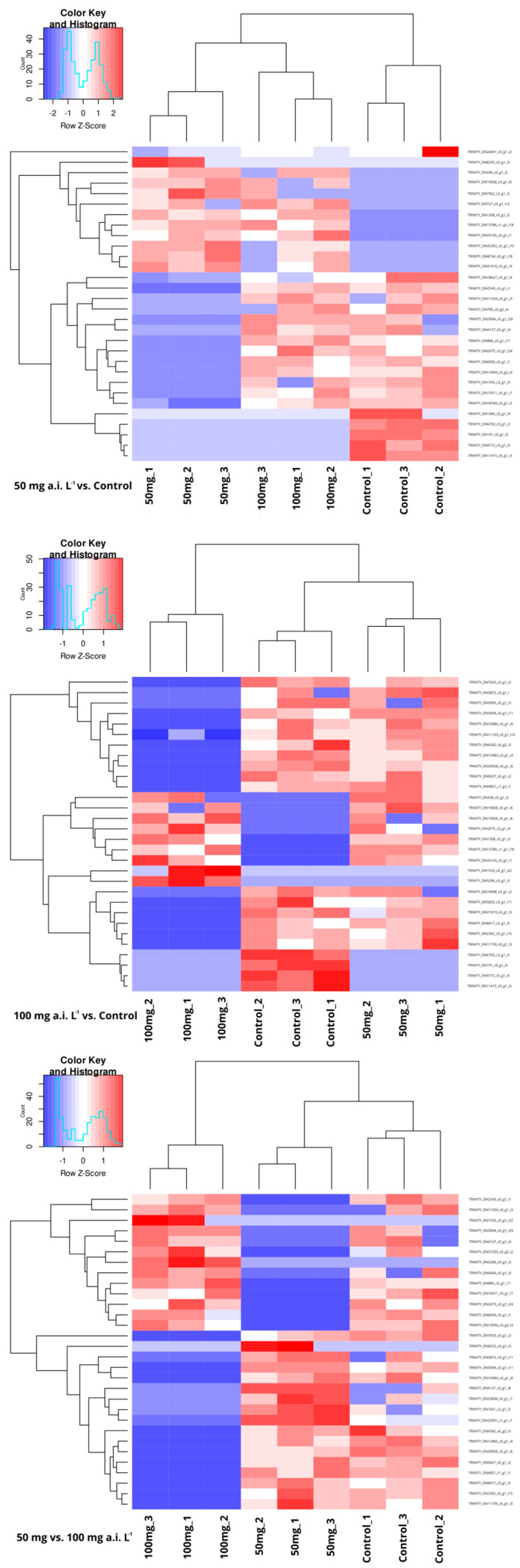
Heatmap analysis of hierarchical clustering of the differentially expressed genes from the testes of *Ceraeochrysa claveri* adults exposed to pyriproxyfen during the larval stage. The heatmap visually represents the expression profiles of all samples using a color gradient, with blue indicating downregulation and red indicating upregulation.

The normalized gene expression data were used to create a heatmap for the differentially expressed genes (DEGs), visually illustrating the expression profiles across all samples, with blue indicating downregulation and red indicating upregulation (Figure 1). A total of 46 DEGs were identified for the 50 mg a.i. L^−1^ treatment compared with the control group (15 upregulated and 31 downregulated). For the 100 mg a.i. L^−1^ treatment versus the control, 47 DEGs were found (16 upregulated and 31 downregulated). Additionally, there were 64 DEGs identified when comparing the 50 mg a.i. L^−1^ and 100 mg a.i. L^−1^ groups (33 upregulated and 31 downregulated) (Table S1).

**Figure 2:**
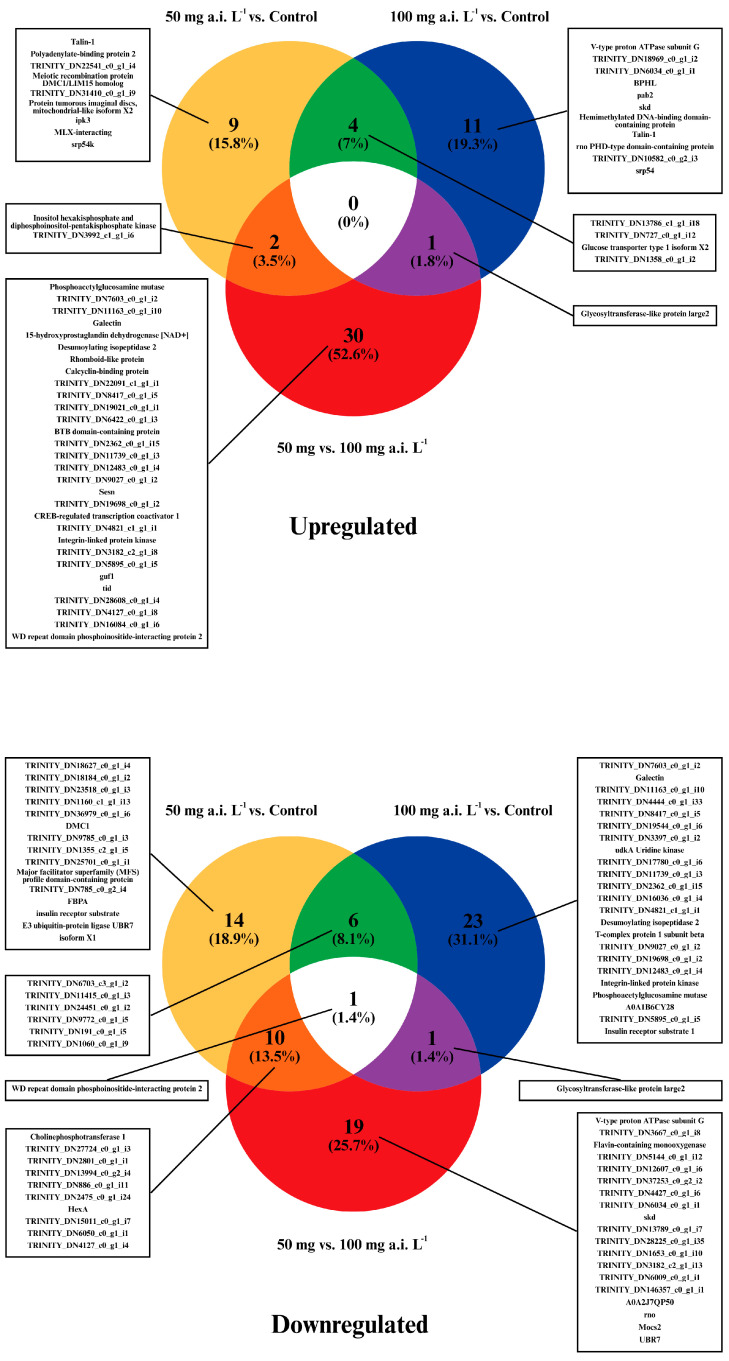
Venn diagrams of the differentially expressed genes (DEGs) of *Ceraeochrysa claveri* testes exposed to pyriproxyfen during the larval stage. The diagrams show the three evaluated groups and the percentages of genes found in all comparisons.

A Venn diagram illustrating the DEGs highlights the three evaluated groups and the percentage of genes common to all comparisons (Figure 2). Gene ontology (GO) analysis of the DEGs revealed key terms within the main GO categories under ‘Biological Processes’ (BP) and ‘Cellular Components’ (CC) ontologies. The most enriched terms for ‘BP’ included Transcription (14.6%), Transport (11.7%), and Cell Cycle (4.4%) (Table S2). In the ‘CC’ category, the top three terms were Membrane (23.5%), Nucleus (18.7%), and Cytoplasm (17.4%) (Table S2). Interestingly, the term ‘Molecular Function’ (MF) was not included, so a manual search for MF associated with each DEG can be found in the Supplementary Materials (Table S3).

### 3.3. RT-qPCR

**Figure 3:**
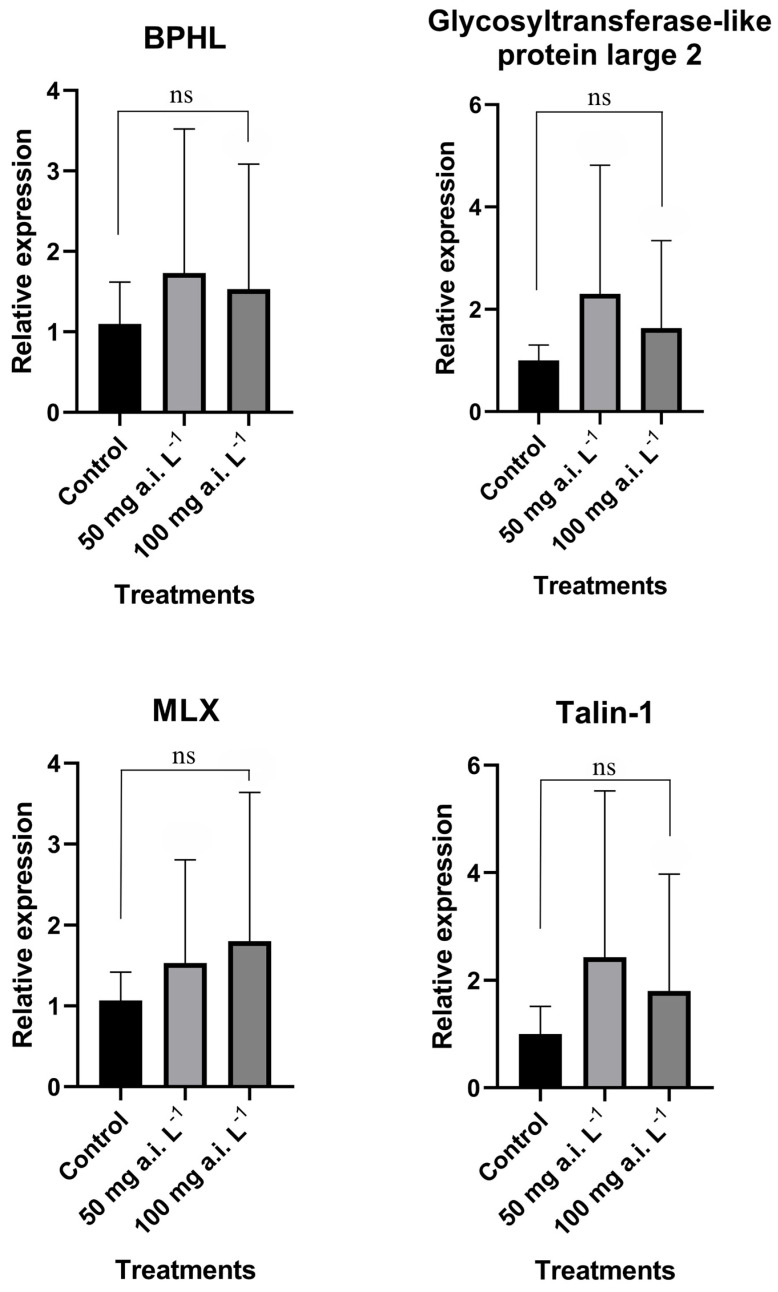
RT-qPCR validation of four differentially expressed genes (DEGs) from the testes of *Ceraeochrysa claveri* adults exposed to two different doses of pyriproxyfen during the larval stage. One-way ANOVA was performed for the comparisons between groups (mean ± SD). ns—not significative.

Four genes were randomly chosen and analyzed to validate the RNA-seq data: Glycosyltransferase-like protein large2, BPHL (Byphenil hydrolase-like), MLX (Max-like protein X)-interacting, and Talin-1. The RT-qPCR fold change results were all upregulated as in the RNA-seq results, indicating that the data were reliable (Figure 3).

## 4. Discussion

Despite exhibiting some cellular damage and ultrastructural alterations in testicular cells [22], this species demonstrated tolerance to the insecticide pyriproxyfen, managing to reproduce and sustain its lifespan under laboratory conditions. This resilience may be attributed to the potential detoxification of the insecticide. To date, there have been no transcriptome data available that show the impact of insecticides on the testes of *C. claveri*. The differentially expressed genes discussed here are reported for the first time in this species.

Byphenyl hydrolase-like (BPHL) was initially identified in human breast cancer [65] and is characterized as a serine hydrolase similar to those that degrade biphenyl compounds in prokaryotes [66,67]. It is highly expressed in the liver and kidneys of mammals, suggesting a role in the detoxification of xenobiotics [65,68]. In *Bombyx mori* (Lepidoptera: Bombycidae), BPHL is predominantly expressed in the fat body and hemolymph, with lower expression levels observed in other tissues, including the testes, where it may play roles in detoxification and immune functions [69]. In our study, BPHL was found to be upregulated in the 100 mg a.i. L^−1^ treatment compared with the control group; however, no significant differences were observed between the 50 mg a.i. L^−1^ treatment and the control or between the 50 mg a.i. L^−1^ and 100 mg a.i. L^−1^ treatments. The testes of *C. claveri* are surrounded by adipose tissue [70], which may facilitate the detoxification of this reproductive organ by enhancing the BPHL expression, thereby ensuring sperm production and viability and supporting offspring production.

LARGE2 is a bifunctional glycosyltransferase (GT) protein that utilizes uridine diphosphate (UDP)–xylose (Xyl) and UDP–glucuronic acid (GlcA) as sugar donors [71,72]. However, it can also accept and donate a variety of sugar ligands, which are implicated in various activities and functionalities, including structural and metabolic functions [73,74]. The biological processes of GTs in insects are crucial for maintaining homeostasis across several metabolic and physiological processes, such as survival, growth, tissue differentiation, and detoxification [75]. Several UDP glycosyltransferases (UGTs) involved in cellular homeostasis have been identified in all insect tissues [76]. The expression of GTs related to detoxification may vary according to the developmental stage of the insect, for instance, this has been observed in *Bombyx mori* during the detoxification of plant allelochemicals [77]. Additionally, GTs may be expressed for specific purposes, such as the detoxification of insecticides, as demonstrated in studies that involved *Plutella xylostella* (Lepidoptera: Plutellidae) [78], *Athetis lepigone* (Lepidoptera: Noctuidae) [79], *Leptinotarsa decemlineata* (Coleoptera: Chrysomelidae) [80], and *Musca domestica* (Diptera: Muscidae) [81]. The RNA-seq results indicate that glycosyltransferase expression was both upregulated (TRINITY_DN29058_c0_g1_I7) and downregulated (TRINITY_DN29058_c0_g1_i6) in the 100 mg a.i. L^−1^ treatment group compared with the control group. This discrepancy may be attributed to our analysis detecting two different isoforms (i6 and i7), with each potentially associated with distinct functions in the testis.

Transcription factors (TFs) play a crucial role in coordinating various biological processes by linking networks associated with diverse metabolic responses [82]. They are essential components in regulating key pathways involved in morphological and developmental cellular processes [83]. The basic helix–loop–helix (bHLH) family is a large group of TFs characterized by their DNA-binding and dimerization domains, which are responsible for development, cell proliferation, and cell lineage determination [84,85]. MLX protein (Max-like protein X) and Max proteins serve as important bHLH TF regulators of cellular metabolism across several model organisms [86,87], with their functions being well conserved between these species [88,89,90]. In mice, the MLX–Mondo complex has various functions, including regulating metabolism to prevent apoptosis and ensuring normal spermatogenesis [91]. Therefore, the upregulation of MLX may indicate an effort to maintain homeostasis. Similar to BPHL, MLX is also found in reproductive tissues [91], and its upregulation may be aimed at supporting the reproduction of *C. claveri* in the field—a desirable trait for this species that helps preserve normal fecundity and fertility.

Talin is a cytoplasmic protein that regulates the interaction between integrin proteins and their ligands, linking them to the actin cytoskeleton through specific binding sites for actin and vinculin [92,93]. In *Drosophila*, studies have demonstrated that Talin enhances the connection between integrins and the extracellular matrix, either by directly linking integrins to the cytoskeleton or by recruiting other actin-binding proteins to facilitate this connection [94,95]. Talin is also associated with cardiomyocyte remodeling, which is essential for maintaining heart contraction and longevity [96]. Additionally, it plays a role in enhancing forces in developing flight muscles [97]. In morphological assays conducted using transmission electron microscopy, testes treated with pyriproxyfen at the same doses used in this study exhibited alterations in the extracellular matrix, including noticeable spacing between cysts [22]. Talin-1 was found to be upregulated in both the 50 mg a.i. L^−1^ vs. control and 100 mg a.i. L^−1^ vs. control treatment groups. Given the reduced expression of integrins ([98] and Table S1), it is possible that Talin-1 was upregulated to help maintain the balance of the cellular ultrastructure.

Over the long term, both the lethal and sublethal effects of insecticides can alter insect diversity in the field, leading to an increase in pest populations and a decline in beneficial insects [99,100,101]. This underscores the importance of integrated pest management (IPM) with new technologies to explore and develop methodologies that combine pesticides with biological control agents [102]. Beneficial insects are affected by insecticides not only during application but also while feeding, resulting in secondary intoxication [101].

Harm effects between insecticides and lacewings have been described. *Chrysoperla genanigra* Freitas (Neuroptera: Chrysopidae) exhibited high larvae mortality when exposed to seven insecticides (azadirachtin, abamectin, cyantraniliprole, thiacloprid, pymetrozine, imidacloprid, and novaluron). Additionally, the surviving adults showed low egg fecundity and fertility [103]. High mortality rates in both larvae and adults were observed in *C. carnea* and *Chrysoperla johnsoni* Henry, Wells and Pupedis (Neuroptera: Chrysopidae) upon exposure to various insecticides. The sublethal effects included reduced survival from larvae to adults and shortened male and female lifespans, as well as decreased fecundity, fertility, and egg viability [104,105]. Similar results—regarding mortality, female fecundity, and egg fertility—were reported when *Chrysoperla externa* Hagen, 1861 (Neuroptera: Chrysopidae) was exposed to acetamiprid + etofenproxy, spinetoram, indoxacarb, and methoxyfenozide [106]. The exposure of *C. claveri* to pyriproxyfen led to increased larvae mortality [23,38] and caused histomorphological alterations in the testes [22], which could result in a decline in *C. claveri* populations and negatively impact its beneficial predation of insect pests.

In this study, all chrysopid adults were survivors of pyriproxyfen exposure during their larval stage, and the findings indicate that the effects of such exposure can persist throughout their adult lifespan, manifesting as alterations in morphology [22] and gene expression, as demonstrated in this research. These results were obtained using RNA sequencing (RNA-seq), a relatively uncommon tool in IPM strategies. Molecular analyses can enhance the approach of IPM in managing pest populations in the field [107], providing genetic data that were previously unavailable for decision-making. Potential discrepancies between the experimental conditions and field applications might end in different results from the same experiment (e.g., insecticide exposure). In the field, direct and indirect contact with insecticides could happen by dermal contact, ingestion, and inhalation [108]. Different from lab conditions, environmental variations, such as abiotic (temperature, humidity, and light changes) and biotic (different diet, vegetation, and crowding population) variations, will change the insect life cycle, physiology, and survival [109], and even with lab results leading to indicators of field performance, further studies involving molecular mechanisms of pyriproxyfen’s disruption of the reproductive–survival balance via testicular gene expression regulation are necessary to solve this issue.

To advance the mechanistic clarity, future studies integrating multi-omics datasets (e.g., proteomics and metabolomics) could map interconnected signaling pathways and reconstruct the regulatory network. As for deeper field exploration, studies comparing the three standard environments [lab, semi-field (cages and greenhouses), and field] [110,111,112] should be performed to enlighten the effects and check whether they persist or disappear when the tested environment changes. Interdisciplinary studies can improve the effectiveness of IPM by reducing the reliance on insecticides, which not only increases profitability (by lowering expenditure on insecticides) but also minimizes the environmental impact of agriculture [113,114], thereby helping to preserve beneficial insect populations.

## Figures and Tables

**Table 1:** Primer sequences for RT-qPCR.

Gene Name	Forward Primer (5′-3′)	Reverse Primer (5′-3′)
BPHL	CCCCACCACTCCATCCTACT	GTTTTCTTGTTGCCGGGTGC
GAPDH	GAACGGGGTCAAGGTAGTGG	AAGTGGTGAAGACTCCGGTT
Glycosyltransferase-like protein large2	GGACTGTATTTGTCATTAAAAAGG	GTTCGTGACCTTTGGTCCATA
MLX-interacting	TAACATGGCTGCTTTGCTTA	ACCTTTGTCACCCGCTGAAT
Talin-1	AACTTCTCGTCCTGCACCT	AATGAAACCGGTCCACTTTG

## Data Availability

The raw data can be accessed on the NCBI website (https://www.ncbi.nlm.nih.gov/sra) under the accession number PRJNA1153524.

## Supplementary Materials

The following supporting information can be downloaded from https://www.mdpi.com/article/10.3390/insects16060567/s1. Table S1: Pyriproxyfen DEGs; Table S2: UniProt search; Table S3: Gene ontology (GO) of the DEGs.
