# Supplementary material for: Intake of Pyriproxyfen Through Contaminated Food by the Predator Ceraeochrysa claveri Navás, 1911 (Neuroptera: Chrysopidae): Evaluation of Long-Term Effects on Testes via Transcriptome Analysis
Source: Insects. 2025 May 28;16(6):567. doi: 10.3390/insects16060567 (PMC12192672; doi:10.3390/insects16060567)
Supplement: Supplementary file 1 [file insects-16-00567-s001.zip › Table S3 - Gene ontology (GO) of the DEGs.pdf]

Gene	GO/ UniProt Code	Biological Process (BP); Molecular Function (MF); Cellular Component (CC)
Inositol hexakisphosphate and diphosphoinositol-pentakisphosphate kinase	GO:0032958<br>GO:0016310<br>GO:0006020	BP: inositol phosphate biosynthetic process; phosphorylation; inositol metabolic process.
	GO:0005524<br>GO:0000827<br>GO:0052723<br>GO:0052724<br>GO:0000829<br>GO:0000828<br>GO:0033857<br>GO:0000832	MF: ATP binding; inositol-1,3,4,5,6-pentakisphosphate kinase activity; inositol hexakisphosphate 1-kinase activity; inositol hexakisphosphate 3-kinase activity; inositol heptakisphosphate kinase activity; inositol hexakisphosphate kinase activity; diphosphoinositol-pentakisphosphate kinase activity; inositol hexakisphosphate 5-kinase activity.
	GO:0005829	CC: cytosol.
Talin 1	GO:0007155<br>GO:0030182<br>GO:0009887<br>GO:0048731	BP: cell adhesion; neuron differentiation; animal organ morphogenesis; system development.
	GO:0004497<br>GO:0016705<br>GO:0003779<br>GO:0005200<br>GO:0051015	MF: monooxygenase activity; oxidoreductase activity, acting on paired donors, with incorporation or reduction of molecular oxygen; actin binding; structural constituent of cytoskeleton; actin filament binding.
	GO:0001726<br>GO:0005737<br>GO:0005856<br>GO:0005925	CC: ruffle; cytoplasm; cytoskeleton; focal adhesion.
Polyadenylate-binding protein 2	GO:0006378	BP: mRNA polyadenylation

Polyadenylate-binding protein 2	GO:0003723<br>GO:0005085<br>GO:0008143	MF: RNA binding; guanyl-nucleotide exchange factor activity; poly(A) binding.
	GO:0005634<br>GO:0055037	CC: nucleus; recycling endosome.
Meiotic recombination protein DMC1/LIM15 homolog	GO:0006302<br>GO:0042771<br>GO:0048477<br>GO:0006268<br>GO:0051106<br>GO:0042148<br>GO:0006310<br>GO:0070192<br>GO:0006281<br>GO:0044671<br>GO:0007127<br>GO:0043066<br>GO:0007294<br>GO:0000724<br>GO:0010569<br>GO:0010032<br>GO:0030717<br>GO:0007143<br>GO:0009949<br>GO:0007131<br>GO:0009792<br>GO:0030716<br>GO:0006312<br>GO:0009951<br>GO:0045003	BP: double-strand break repair; intrinsic apoptotic signaling pathway in response to DNA damage by p53 class mediator; oogenesis; DNA unwinding involved in DNA replication; positive regulation of DNA ligation; strand invasion; DNA recombination; chromosome organization involved in meiotic cell cycle; DNA repair; sorocarp spore cell differentiation; meiosis I; negative regulation of apoptotic process; germarium-derived oocyte fate determination; double-strand break repair via homologous recombination; regulation of double-strand break repair via homologous recombination; meiotic chromosome condensation; oocyte karyosome formation; female meiotic nuclear division; polarity specification of anterior/posterior axis; reciprocal meiotic recombination; embryo development ending in birth or egg hatching; oocyte fate determination; mitotic recombination; polarity specification of dorsal/ventral axis; double-strand break repair via synthesis-dependent strand annealing; cell motility; mitotic recombination-dependent replication fork processing; DNA recombinase assembly; intracellular mRNA localization.

Meiotic recombination protein DMC1/LIM15 homolog	GO:0048870<br>GO:1990426<br>GO:0000730<br>GO:0008298	
	GO:0003690<br>GO:0003697<br>GO:0000150<br>GO:0042802<br>GO:0140664<br>GO:0017116<br>GO:0017151<br>GO:0005524<br>GO:0008094	MF: double-stranded DNA binding; single-stranded DNA binding; DNA strand exchange activity; identical protein binding; ATP-dependent DNA damage sensor activity; single-stranded DNA helicase activity; DEAD/H-box RNA helicase binding; ATP binding; ATP-dependent activity, acting on DNA.
	GO:0043073<br>GO:0005634<br>GO:0000794<br>GO:0000785<br>GO:0035861	CC: germ cell nucleus; nucleus; condensed nuclear chromosome; chromatin; site of double-strand break.
ipk3	GO:0032958<br>GO:0016310	BP: inositol phosphate biosynthetic process; phosphorylation.
	GO:0051765<br>GO:0016301<br>GO:0008440	MF: inositol tetrakisphosphate kinase activity; kinase activity; inositol-1,4,5-trisphosphate 3-kinase activity.
	A0A2J7Q4N6	CC: cytoplasm; nucleus.
tid	GO:0002119<br>GO:0006457<br>GO:0009792<br>GO:0045879<br>GO:0009408<br>GO:0007005	BP: nematode larval development; protein folding; embryo development ending in birth or egg hatching; negative regulation of smoothened signaling pathway; response to heat; mitochondrion organization; chaperone-mediated protein folding; negative regulation of apoptotic process; chromatin organization.

tid	GO:0061077<br>GO:0043066<br>GO:0006325	
	GO:0005113<br>GO:0005524<br>GO:0051082<br>GO:0030544<br>GO:0031072<br>GO:0046872	MF: patched binding; ATP binding; unfolded protein binding; Hsp70 protein binding; heat shock protein binding; metal ion binding.
	GO:0005829<br>GO:0005739<br>GO:0005694<br>GO:0005741	CC: cytosol ;mitochondrion; chromosome; mitochondrial outer membrane.
MLX-interacting	GO:0070328<br>GO:0042593<br>GO:0009744	BP: triglyceride homeostasis; glucose homeostasis; response to sucrose.
	GO:0046983<br>A0A8K0GJF7	MF: protein dimerization activity; DNA-binding transcription factor activity, RNA polymerase II-specific; RNA polymerase II cis-regulatory region sequence-specific DNA binding.
	A0A8K0GJF7	CC: cytoplasm; nucleus.
srp54k	GO:0006616<br>GO:0006614	BP: SRP-dependent cotranslational protein targeting to membrane, translocation; SRP-dependent cotranslational protein targeting to membrane.
	GO:0008312<br>GO:0003924<br>GO:0030942<br>GO:0005525	MF: 7S RNA binding; GTPase activity; endoplasmic reticulum signal peptide binding; GTP binding.

srp54k	GO:0005783<br>GO:0005786<br>GO:0048500<br>GO:0016607<br>GO:0045335	CC: endoplasmic reticulum; signal recognition particle, endoplasmic reticulum targeting; signal recognition particle; nuclear speck; phagocytic vesicle.
Cholinephosphotransferase 1	GO:0008654	BP: Phospholipid biosynthetic process;
	GO:0016780<br>GO:0016747	MF: phosphotransferase activity, for other substituted phosphate groups; acyltransferase activity, transferring groups other than amino-acyl groups.
	GO:0016020	CC: Membrane.
Meiotic recombination protein dmc1	GO:0009949<br>GO:0007143<br>GO:0030717<br>GO:0010032<br>GO:0009951<br>GO:0045003<br>GO:0006312<br>GO:0030716<br>GO:0009792<br>GO:0007131<br>GO:0000730<br>GO:1990426<br>GO:0048870<br>GO:0008298<br>GO:0006302<br>GO:0006268<br>GO:0051106<br>GO:0042771<br>GO:0048477<br>GO:0006281	BP: polarity specification of anterior/posterior axis; female meiotic nuclear division; oocyte karyosome formation; meiotic chromosome condensation; polarity specification of dorsal/ventral axis; double-strand break repair via synthesis-dependent strand annealing; mitotic recombination; oocyte fate determination; embryo development ending in birth or egg hatching; reciprocal meiotic recombination;DNA recombinase assembly; mitotic recombination-dependent replication fork processing; cell motility; intracellular mRNA localization; double-strand break repair; DNA unwinding involved in DNA replication; positive regulation of DNA ligation; intrinsic apoptotic signaling pathway in response to DNA damage by p53 class mediator; oogenesis; DNA repair; sorocarp spore cell differentiation; chromosome organization involved in meiotic cell cycle; DNA recombination; strand invasion; regulation of double-strand break repair via homologous recombination; double-strand break repair via homologous recombination; germarium-derived oocyte fate determination; negative regulation of apoptotic process; meiosis I.

	GO:0044671<br>GO:0070192<br>GO:0006310<br>GO:0042148<br>GO:0010569<br>GO:0000724<br>GO:0007294<br>GO:0043066<br>GO:0007127	
Meiotic recombination protein dmc1	GO:0017116<br>GO:0140664<br>GO:0005524<br>GO:0017151<br>GO:0003677<br>GO:0008094<br>GO:0003690<br>GO:0003697<br>GO:0042802<br>GO:0000150	MF: single-stranded DNA helicase activity; ATP-dependent DNA damage sensor activity; ATP binding; DEAD/H-box RNA helicase binding; DNA binding; ATP-dependent activity, acting on DNA; double-stranded DNA binding; single-stranded DNA binding; identical protein binding; DNA strand exchange activity.
	GO:0000785<br>GO:0035861<br>GO:0043073<br>GO:0000794<br>GO:0005634	CC: chromatin; site of double-strand break; germ cell nucleus; condensed nuclear chromosome; nucleus.
Major facilitator superfamily (MFS) profile domain-containing protein	A0A834IJA8 (UniProt code)	MF: sugar transmembrane transporter activity.
		CC: plasma membrane.
HexA	GO:0045735<br>GO:0005615<br>GO:0005616	MF: nutrient reservoir activity.<br>CC: extracellular space; larval serum protein complex.

WD repeat domain phosphoinositide-interacting protein 2	GO:0000422<br>GO:0044804<br>GO:0010506<br>GO:0009267<br>GO:0061365<br>GO:0098792<br>GO:0010508<br>GO:0036093<br>GO:0043277<br>GO:0009792<br>GO:0061723<br>GO:0006497<br>GO:0051607<br>GO:0040024<br>GO:0035096<br>GO:0001778<br>GO:0034497<br>GO:0008340<br>GO:0012501<br>GO:0006914<br>GO:0097237<br>GO:0000045<br>GO:0042078<br>GO:0050688<br>GO:0048598<br>GO:0014067<br>GO:0030163	BP: autophagy of mitochondrion; autophagy of nucleus; regulation of autophagy; cellular response to starvation; positive regulation of triglyceride lipase activity; xenophagy; positive regulation of autophagy; germ cell proliferation; apoptotic cell clearance; embryo development ending in birth or egg hatching; glycophagy; protein lipidation; defense response to virus; dauer larval development; larval midgut cell programmed cell death; plasma membrane repair; protein localization to phagophore assembly site; determination of adult lifespan; programmed cell death; autophagy; cellular response to toxic substance; autophagosome assembly; germ-line stem cell division; regulation of defense response to virus; embryonic morphogenesis; negative regulation of phosphatidylinositol 3-kinase signaling; protein catabolic process.
	GO:0080025<br>GO:0070273	MF: phosphatidylinositol-3,5-bisphosphate binding; phosphatidylinositol-4-phosphate binding; phosphatidylinositol-5-phosphate binding; phosphatidylinositol-3-phosphate binding.

WD repeat domain phosphoinositide-interacting protein 2	GO:0010314<br>GO:0032266	
	GO:0019898<br>GO:0034045<br>GO:0045335<br>GO:0005776<br>GO:0030670	CC: extrinsic component of membrane; phagophore assembly site membrane; phagocytic vesicle; autophagosome; phagocytic vesicle membrane.
Fructose-biphosphate aldolase	GO:0008154<br>GO:0007498<br>GO:0006000<br>GO:0006096<br>GO:0042593<br>GO:0051289<br>GO:0030388	BP: actin polymerization or depolymerization; mesoderm development; fructose metabolic process; glycolytic process; glucose homeostasis; protein homotetramerization; fructose 1,6-bisphosphate metabolic process.
	GO:0042802<br>GO:0004332<br>GO:0003779	MF: identical protein binding; fructose-bisphosphate aldolase activity; actin binding.
	GO:0016020<br>GO:0045335<br>GO:0055120<br>GO:0031012<br>GO:0030018<br>GO:0005829<br>GO:0020015<br>GO:0000792<br>GO:0005737<br>GO:0020002<br>GO:0031430<br>GO:0030017	CC: membrane; phagocytic vesicle; striated muscle dense body; extracellular matrix; Z disc; cytosol; glycosome; heterochromatin; cytoplasm; host cell plasma membrane; M band; sarcomere.

Insulin receptor substrate 1	GO:0010897<br>GO:0042594<br>GO:0035264<br>GO:0007568<br>GO:0014068<br>GO:0008286<br>GO:0060250<br>GO:0007285<br>GO:0045793<br>GO:0009267<br>GO:0035159<br>GO:0040018<br>GO:0008284<br>GO:0034059<br>GO:1903688<br>GO:0060291<br>GO:0061964<br>GO:0045927<br>GO:0046622<br>GO:0048133<br>GO:0048477<br>GO:0008355<br>GO:0050778<br>GO:0007296<br>GO:0007295<br>GO:0048009<br>GO:0042593<br>GO:0055088<br>GO:0043491<br>GO:0008340	BP: negative regulation of triglyceride catabolic process; response to starvation; multicellular organism growth; aging; positive regulation of phosphatidylinositol 3-kinase signaling; insulin receptor signaling pathway; germ-line stem-cell niche homeostasis; primary spermatocyte growth; positive regulation of cell size; cellular response to starvation; regulation of tube length, open tracheal system; positive regulation of multicellular organism growth; positive regulation of cell population proliferation; response to anoxia; positive regulation of border follicle cell migration; long-term synaptic potentiation; negative regulation of entry into reproductive diapause; positive regulation of growth; positive regulation of organ growth; male germ-line stem cell asymmetric division; oogenesis; olfactory learning; positive regulation of immune response; vitellogenesis; growth of a germarium-derived egg chamber; insulin-like growth factor receptor signaling pathway; glucose homeostasis; lipid homeostasis; protein kinase B signaling; determination of adult lifespan.

	GO:0043548<br>GO:0005158<br>GO:0042169<br>GO:0005159	MF: phosphatidylinositol 3-kinase binding; insulin receptor binding; SH2 domain binding; insulin-like growth factor receptor binding.
	GO:0005938<br>GO:0005829<br>GO:0043231	CC: cell cortex; cytosol; intracellular membrane-bounded organelle.
E3 ubiquitin-protein ligase UBR7	GO:0008270<br>GO:0016874<br>GO:0061630	MF: ubiquitin protein ligase activity; zinc ion binding; ligase activity.
	A0A6J0B309	CC: cytoplasm.
V-type proton ATPase subunit G	E0VSA0	MF: proton-transporting ATPase activity; rotational mechanism.
		CC: vacuolar proton-transporting V-type ATPase complex.
Glycosyltransferase-like protein large2	A0A7R8VUK5	BP: protein O-linked mannolysation
	GO:0016757<br>A0A7R8VUK5	MF: glycosyltransferase activity; glucuronosyltransferase activity; xylosyltransferase activity.
	GO:0016020<br>A0A7R8VUK5	CC: membrane; Golgi apparatus.
BPHL	GO:0016787	MF: hydrolase activity
pab2	GO:0006378	BP: mRNA polyadenylation
	GO:0003723<br>GO:0005085<br>GO:0008143	MF: RNA binding; guanyl-nucleotide exchange factor activity; poly(A) binding.
	GO:0005634<br>GO:0055037	CC: nucleus; recycling endosome.
skd	GO:0006367<br>GO:0036011<br>GO:0048749<br>GO:0048190	BP: transcription initiation at RNA polymerase II promoter; imaginal disc-derived leg segmentation; compound eye development; wing disc dorsal/ventral pattern formation; cell fate commitment; sex comb development; regulation of transcription by RNA polymerase II;

skd	GO:0045165<br>GO:0045498<br>GO:0006357<br>GO:0045944<br>GO:0007526<br>GO:0022416<br>GO:0090263	positive regulation of transcription by RNA polymerase II; larval somatic muscle development; chaeta development; positive regulation of canonical Wnt signaling pathway.
	GO:0003712<br>GO:0003713	MF: transcription coregulator activity; transcription coactivator activity.
	GO:1990904<br>GO:0005654<br>GO:0016592<br>GO:0070847<br>GO:0005840	CC: ribonucleoprotein complex; nucleoplasm; mediator complex; core mediator complex; ribosome.
Hemimethylated DNA-binding domain-containing protein	A0A8K0C9Y2	MF: DNA binding
PHD-type domain-containing protein	GO:0006325<br>GO:0042051<br>GO:0035194<br>GO:0034729<br>GO:0006357	BP: chromatin organization; compound eye photoreceptor development; ncRNA-mediated post-transcriptional gene silencing; histone H3-K79 methylation; regulation of transcription by RNA polymerase II.
	GO:0035064<br>GO:0003682<br>GO:0031491<br>GO:0010698<br>GO:0046872<br>GO:0042393	MF: methylated histone binding; chromatin binding; nucleosome binding; acetyltransferase activator activity; metal ion binding; histone binding.
	GO:0000793<br>GO:0005694<br>GO:0005634	CC: condensed chromosome; chromosome;nucleus; histone methyltransferase complex; MOZ/MORF histone acetyltransferase complex; chromatin.

	GO:0035097<br>GO:0070776<br>GO:0000785	
Glucose transporter type 1	GO:0015749<br>GO:0090277<br>GO:0008643<br>GO:1904659<br>GO:0042593	BP: monosaccharide transmembrane transport; positive regulation of peptide hormone secretion; carbohydrate transport; glucose transmembrane transport; glucose homeostasis.
	GO:0015149<br>GO:0022857	MF: hexose transmembrane transporter activity; transmembrane transporter activity.
	GO:0016020<br>GO:0016323	CC: membrane; basolateral plasma membrane.
Galectin	A0A6J2X3K1	MF: Carbohydrate binding;
udkA Uridine kinase	GO:0044206<br>GO:0008655<br>GO:1901564<br>GO:0044211<br>GO:0016310<br>GO:0044238<br>GO:0009792	BP: UMP salvage; pyrimidine-containing compound salvage; organonitrogen compound metabolic process; CTP salvage; phosphorylation; primary metabolic process; embryo development ending in birth or egg hatching.
	GO:0004849<br>GO:0016301<br>GO:0043771<br>GO:0004845<br>GO:0005524	MF: uridine kinase activity; kinase activity; cytidine kinase activity; uracil phosphoribosyltransferase activity; ATP binding.
	GO:0022625	CC: cytosolic large ribosomal subunit.
Desumoylating isopeptidase 2	A0A6P4J309	MF: Deubiquitinase activity.
T-complex protein 1 subunit beta	GO:0006457	BP: protein folding
	GO:0005524<br>GO:0140662	MF: ATP binding; ATP-dependent protein folding chaperone; ATP hydrolysis activity; unfolded protein binding.

T-complex protein 1 subunit beta	GO:0016887<br>GO:0051082	
	GO:0045335<br>GO:0005832	CC: phagocytic vesicle;chaperonin-containing T-complex.
Integrin-linked protein kinase	GO:0006887<br>GO:0007160<br>GO:0055002<br>GO:0034446<br>GO:0032956<br>GO:1904951<br>GO:0040017<br>GO:1904901<br>GO:0046716<br>GO:0032259<br>GO:0030239<br>GO:1903829<br>GO:0007005<br>GO:0006468<br>GO:0007229<br>GO:0009792<br>GO:0060298	BP: exocytosis; cell-matrix adhesion; striated muscle cell development; substrate adhesion-dependent cell spreading; regulation of actin cytoskeleton organization; positive regulation of establishment of protein localization; positive regulation of locomotion; positive regulation of myosin II filament organization; muscle cell cellular homeostasis; methylation; myofibril assembly; positive regulation of protein localization; mitochondrion organization; protein phosphorylation; integrin-mediated signaling pathway; embryo development ending in birth or egg hatching; positive regulation of sarcomere organization.
	GO:0008168<br>GO:0004672<br>GO:0016301<br>GO:0030674<br>GO:0005524<br>GO:0005178	MF: methyltransferase activity; protein kinase activity; kinase activity; protein-macromolecule adaptor activity; ATP binding; integrin binding.
	GO:0008305<br>GO:0009925<br>GO:0005912	CC: integrin complex; basal plasma membrane; adherens junction; striated muscle dense body; host cell presynaptic membrane; other organism cell membrane; focal adhesion; M band.

Integrin-linked protein kinase	GO:0055120<br>GO:0044231<br>GO:0044218<br>GO:0005925<br>GO:0031430	
Phosphoacetylglucosamine mutase	GO:0006048<br>GO:0016539<br>GO:0016540<br>GO:0048731<br>GO:0005975<br>GO:0007267	BP: UDP-N-acetylglucosamine biosynthetic process; intein-mediated protein splicing; protein autoprocessing; system development; carbohydrate metabolic process; cell-cell signaling.
	GO:0000287<br>GO:0004610<br>GO:0016868	MF: magnesium ion binding; phosphoacetylglucosamine mutase activity; intramolecular transferase activity, phosphotransferases.
15-hydroxyprostaglandin dehydrogenase [NAD+]	A0A140KPR8	MF: oxidoreductase activity; acting on the CH-OH group of donors; NAD or NADP as acceptor.
		CC: cytoplasm.
Rhomboid-like protein	A0A1B0FHL1	MF: Serine-type endopeptidase activity.
		CC: Membrane
Calcyclin-binding protein	A0A6P7G295	BP: heart development
		MF: S100 protein binding; tubulin binding; ubiquitin protein ligase binding.
		CC: cytoplasm; nucleus.
BTB domain-containing protein	GO:0030431<br>GO:0071805<br>GO:0006813<br>GO:0034220<br>GO:0034765<br>GO:0051260	BP: sleep; potassium ion transmembrane transport; potassium ion transport; monoatomic ion transmembrane transport; regulation of monoatomic ion transmembrane transport; protein homooligomerization.
	GO:0005251<br>GO:0005249<br>GO:0022843	MF: delayed rectifier potassium channel activity; voltage-gated potassium channel activity; voltage-gated monoatomic cation channel activity; voltage-gated monoatomic ion channel activity.

BTB domain-containing protein	GO:0005244	
	GO:0032809<br>GO:0032590<br>GO:0030424<br>GO:0008076	CC: neuronal cell body membrane; dendrite membrane; axon; voltage-gated potassium channel complex.
Sesn	GO:1901031<br>GO:0030308<br>GO:1904262<br>GO:0016239<br>GO:1990253<br>GO:0000422<br>GO:0071233<br>GO:2000377<br>GO:0010259	BP: regulation of response to reactive oxygen species; negative regulation of cell growth; negative regulation of TORC1 signaling; positive regulation of macroautophagy; cellular response to leucine starvation; autophagy of mitochondrion; cellular response to leucine; regulation of reactive oxygen species metabolic process; multicellular organism aging.
	GO:0070728<br>GO:0016684<br>GO:0004601	MF: leucine binding; oxidoreductase activity, acting on peroxide as acceptor; peroxidase activity.
	GO:0005737<br>GO:0005634	CC: cytoplasm; nucleus.
CREB-regulated transcription coactivator 1	M9PFU2	BP: cellular responde to cAMP; positive regulation of DNA-templated transcription; positive regulation of transcription by RNA polymerase II; protein homotetramerization; responde to oxidative stress; response to starvation.
		MF:cAMP response element binding protein binding.
		CC: cytoplasm; cytosol; nucleus.
guf1	GO:0045727<br>GO:0006412	BP: positive regulation of translation; translation.
guf1	GO:0043022<br>GO:0097177<br>GO:0005525<br>GO:0003924	MF: ribosome binding; mitochondrial ribosome binding; GTP binding; GTPase activity.

	GO:0005739<br>GO:0005759<br>GO:0005743	CC: mitochondrion; mitochondrial matrix; mitochondrial inner membrane.
Flavin-containing monooxygenase	GO:0050660<br>GO:0004499<br>GO:0050661	MF: Flavin adenine dinucleotide binding; N,N-dimethylaniline monooxygenase activity; NADP binding;
Covalently-linked cell wall protein	A0A1Y1L5D7	BP: fungal-type cell wall organization
Mocs2	GO:0006139<br>GO:0006338<br>GO:0006777<br>GO:0006807<br>GO:0032324<br>GO:0043605	BP: nucleobase-containing compound metabolic process; chromatin remodeling; Mo-molybdopterin cofactor biosynthetic process; nitrogen compound metabolic process; molybdopterin cofactor biosynthetic process; amide catabolic process.
	GO:0000166<br>GO:0016811<br>GO:0030366<br>GO:0047710<br>GO:0110050	MF: nucleotide binding; hydrolase activity, acting on carbon-nitrogen (but not peptide) bonds, in linear amides; molybdopterin synthase activity; bis(5'-adenosyl)-triphosphatase activity; deaminated glutathione amidase activity.
	GO:0005700<br>GO:0005829<br>GO:0019008<br>GO:0140672	CC: polytene chromosome; cytosol; molybdopterin synthase complex; ATAC complex.
